# A retrospective, Italian multicenter study of complex abdominal wall defect repair with a Permacol biological mesh

**DOI:** 10.1038/s41598-020-60019-0

**Published:** 2020-02-25

**Authors:** Domenico Russello, Maria Sofia, Piero Conti, Saverio Latteri, Antonino Pesce, Francesco Scaravilli, Fabio Vasta, Giovanni Trombatore, Valentina Randazzo, Elena Schembari, Martina Barchitta, Antonella Agodi, Gaetano La Greca

**Affiliations:** 1 General Surgery, Cannizzaro Hospital, Catania, Italy; 2 Department of Medical, Surgical Sciences and Advanced Technologies “G.F. Ingrassia”, University of Catania, Catania, Italy; 3 General Surgery, Civil Hospital, Lentini, Italy; 4 General Surgery, “Policlino-Vittorio Emanuele” Hospital, Catania, Italy; 5 General Surgery, “San Vincenzo” Hospital, Taormina, Italy

**Keywords:** Anatomy, Gastroenterology, Digestive signs and symptoms

## Abstract

Complex abdominal wall defects (CAWDs) can be difficult to repair and using a conventional synthetic mesh is often unsuitable. A biological mesh might offer a solution for CAWD repair, but the clinical outcomes are unclear. Here, we evaluated the efficacy of a cross-linked, acellular porcine dermal collagen matrix implant (Permacol) for CAWD repair in a cohort of 60 patients. Here, 58.3% patients presented with a grade 3 hernia (according to the Ventral Hernia Working Group grading system) and a contaminated surgical field. Permacol was implanted as a bridge in 46.7%, as an underlay (intraperitoneal position) in 38.3% and as a sublay (retromuscolar position) in 15% of patients. Fascia closure was achieved in 53.3% of patients. The surgical site occurrence rate was 35% and the defect size significantly influenced the probability of post-operative complications. The long-term (2 year) hernia recurrence rate was 36.2%. This study represents the first large multi-centre Italian case series on Permacol implants in patients with a CAWD. Our data suggest that Permacol is a feasible strategy to repair a CAWD, with acceptable early complications and long-term (2 year) recurrence rates.

## Introduction

Incisional hernias represent a common complication of abdominal wall surgery, with an incidence of 11–20%^1^. Direct surgical repair has an estimated failure rate of 49–63%^2,3^. The use of a synthetic mesh for repair has significantly reduced this recurrence rate to 10–30%^2,3^; however, this reduction is counterbalanced by a potential increased risk of infection as a result of the implanted synthetic material^3^. Overall, a synthetic mesh is generally thought to be safe and effective for standard hernia repair^2^, where it has a low associated risk of complications. Whether this consensus is true for patients with a complex abdominal wall defect (CAWD), however, is unclear.

A consensus meeting involving a large expert panel defined CAWD as any defect presenting with two or more of the following criteria: large or multiple abdominal defects ≥10 cm in width; wound class III (contaminated) or IV (dirty); parastomal, lumbar or subcostal location; multiple previous repair; loss of domain; presence of fistula; open abdomen; and/or no primary closure possible without component separation technique^4,5^. Although CAWD repair represents a surgical challenge, the use of a mesh is currently mandatory^2^; the exception is for cases where the CAWD shows a contaminated/infected field. Here, a synthetic mesh is not recommended due to its association with severe post-operative complications^6^.

A biological mesh is an alternative surgical material to a synthetic mesh; here, the mesh incorporates into the native tissue and is less susceptible to causing an infection^1,6,7^. Many different types of biological mesh are available, including bovine, porcine and human implants. Permacol is an acellular porcine-derived collagen matrix graft that is cross-linked through hexamethylene diisocyanate. The cross-linking process supports fibroblast growth and provides resistance against collagenase enzymes. The inability of collagenase to digest the implant allows it to maintain its structural integrity; the mesh is, however, degraded over time^8–10^.

Over recent years, there has been an increasing trend toward using a biological mesh in contaminated/infected surgical fields and for CAWD. The surgical management of patients with CAWD varies, and sometimes tailored individual patient care is required. While some favorable, short-term and long-term outcomes after biological mesh repair have been reported^5,11–16^, the wide range of variables affecting surgical management has resulted in conflicting patient outcomes. These inconsistencies cast doubt on the utility of biologic materials for CAWD repair^17–22^.

The present study was motivated by the relative paucity of large studies analyzing clinical outcomes after CAWD repair. Here, we evaluated the clinical outcomes following Permacol implantation for CAWD repair in a cohort of 60 patients.

## Methods

### Study approval and patient consent

All patients provided informed consent prior to surgery using a xenogenic porcine-derived biologic mesh for CAWD repair. All methods were carried out in accordance with the relevant guidelines and the regulations of the Declaration of Helsinki. This retrospective study was approved by the Ethics Committee of the University of Catania, Italy.

### Study design and setting

This retrospective multicenter study included patients with CAWD who underwent abdominal wall reconstruction with Permacol between January 2010 and May 2016 at four public hospitals in Italy: “Cannizzaro” Hospital, Catania; Civil Hospital, Lentini; “Policlinico-Vittorio Emanuele” Hospital, Catania; and “San Vincenzo” Hospital, Taormina. Each hospital received a questionnaire to complete regarding the preoperative data, surgical details and clinical outcomes for each patient. The patient eligibility criteria for CAWD were as previously described^4,5^. The data collected from individual medical records included: patient demographics (age, gender), body mass index (BMI), ASA score, co-morbidities, medical history of abdominal neoplasms, previous abdominal wall surgery, wound morbidity classification [modified Ventral Hernia Working Group (VHWG) grade]^23^, hernia Chevrel classification^24^, indication to surgery, surgical technique, mesh size, operation time, morbidity, length of hospital stay and hernia recurrence rate. All data were entered into an online database and then analyzed.

### Pre-operative, operative and post-operative procedures

All patients underwent a pre-operative clinical assessment and the CAWD was evaluated by computed tomography (CT). The CT images were used to identify the CAWD site, the width and number of the defects, the anatomical position and trophic status of the rectus abdominis muscles, loss of domain, fistula and the presence of any other wounds or complications. Loss of domain was defined when the herniated organs lost their right of domain inside the abdominal cavity and so the volume of the hernia could no longer be reduced to the abdominal cavity.

All patients were operated on under general anesthesia and were given perioperative antibiotics according to hospital protocol. In each of the four hospitals, the same surgeon performed the CAWD repair for all patients. The surgeons across all four hospitals used one of three main surgical techniques to close the defect with the Permacol implant: (1) sublay, placement of the biologic mesh in the retromuscolar space in an extra-peritoneal position after the closure of the rectus posterior fascia; (2) underlay, placement of the mesh in an intraperitoneal position deep to anterior abdominal wall defect and then closure of the fascia over the mesh; or (3) bridge, placement of the mesh in an intraperitoneal position, with the center part of the mesh forming a bridge between the edges of the rectus sheath when fascial closure was not feasible. All patients attended 3, 6, 12 and 24-month post-operative follow-up examinations with the original surgeon. All patients were assessed by abdominal examination, and patients with clinical suspicion of recurrence were also assessed by CT to rule out recurrence.

### Study outcomes

The study outcomes were as follows: (1) surgical site occurrence (SSO) at 30 postoperative days; and (2) recurrence rate at 1-year and 2-years follow-up. SSO was defined as the presence of one or more of the following features: wound dehiscence, skin/fat necrosis, cellulitis/abscess, hematoma, seroma and/or enterocutaneous fistula. Recurrence was defined as a fascial defect at the surgical site. Bulging was defined as a relaxing of the abdominal wall at the mesh site due to stretching of the mesh, without any interruption between the mesh and the abdominal wall layer^21,25^. Bulging was considered in the SSO group and was not considered as a recurrence. The complications were defined according to the Clavien-Dindo classification system^26^.

### Statistical analyses

All collected data were reported in a specific study database and statistical analyses were performed using SPSS software (IBM SPSS Statistics for Windows, version 23.0). Descriptive statistics were used to characterize the study population by using frequencies, mean and median values. A two-tailed Chi-square test was used to statistically compare proportions. Continuous variables were tested for normality using the Kolmogorov-Smirnov test and compared by Student’s t-test or Mann-Whitney test for independent samples, as appropriate. A *p*-value < 0.05 was considered statistically significant.

### Informed consent

Informed consent was obtained from all individual participants included in the study.

## Results

### Cohort characteristics

We recruited 60 eligible patients from four Italian hospitals who underwent wall reconstruction with Permacol implantation for a CAWD between January 2010 and May 2016 (Table 1). Most patients underwent Permacol implantation at the “Cannizzaro” (n = 21) or “Policlinico-Vittorio Emanuele” (n = 24) hospitals. The remaining patients were treated at the Civil Hospital of Lentini (n = 5) or the “San Vincenzo” hospital (n = 10). The mean age was 62 years (range 41–88 years) with 39 (65%) females and 21 (35.5%) males. The ASA score was high (III-IV) in 63.3% of patients. Sixteen patients (26.6%) had a BMI >35, such as 16 (26.6%) patients had diabetes and 16 (26.6%) patients had pulmonary disease; 22 (36.6%) patients were smokers. Abdominal surgery for neoplastic disease had been previously performed in 43.3% of the patients (Table 1)

**Table 1:** Patient demographics and preoperative data.

**Total number of patients**	60
**Mean age (years)**	62 (range 41–88)
**Gender (male/female)**	21/39
**Co-morbidities**	
BMI > 35	16 (26.7%)
Diabetes mellitus	16 (26.7%)
COPD	16 (26.7%)
Smoking status	22 (36.7%)
**Previous neoplastic surgery**	26 (43.3%)
Colorectal cancer	14 (53.8%)
gynecological tumors	4 (15.4%)
urological tumors	4 (15.4%)
other neoplasms	4 (15.4%)
**ASA score**	
ASA I	—
ASA II	22 (36.7%)
ASA III	26 (43.3%)
ASA IV	12 (20%)
**Chevrel classification**	
M1	10 (16.6%)
M2	13 (21.6%)
M3	10 (16.6%)
M4	25 (41.6%)
L	2 (3.3%)
W1	2 (3.3%)
W2	12 (20%)
W3	22 (36.6%)
W4	23 (38.3%)
**VHWG grade**	
Grade 1	4 (6.7%)
Grade 2	21 (35%)
Grade 3	35 (58.3%)

BMI: body mass index; COPD: Chronic Obstructive Pulmonary Disease; ASA: American Society of Anesthesiologists; VHWG: modified Ventral Hernia Working Group;.

### Defect characteristics

The abdominal wall defect was categorized as a recurrent incisional hernia in 12 (20%) patients: six cases were the first recurrence, two cases were the second recurrence, three cases were the third recurrence and one case was the fourth recurrence. Most (96.7%) defects were located along the midline, with only two cases (3.3%) being lateral. According to the Chevrel classification, the midline defects were predominantly M3-M4 and W3-W4 (Table 1).

### Surgical indications

Complex incisional hernias were identified in 58 (96.6%) patients; the remaining two patients presented with a parastomal hernia or an open abdomen. The complex incisional hernias included: 45 (75%) defects >10 cm wide, 14 (23.3%) defects with stoma presence requiring a surgical stoma closure procedure, 13 (21.6%) hernias with loss of domain, 12 (20%) defects with an infected synthetic mesh, seven (11.6%) cutaneous/enteric fistulas and seven (11.6%) surgical wound infections. According to the modified VHWG grading system^23^, 6.6% hernias were categorized as grade 1, 35% as grade 2 and 58.3% as grade 3.

### Surgical procedure

Surgery was performed as an emergency in 20% of cases. Permacol was implanted in the sublay position in nine (15%) patients, in the underlay position in 23 (38.3%) patients and as a bridge in 28 (46.7%) patients. Fascial closure was achieved in 53.3% of the surgical procedures. The median mesh size was 540 cm^2^ (range 120-1,400 cm^2^), and in three patients two Permacol meshes were implanted to ensure comprehensive coverage of the defect. In three other patients, a partially absorbable synthetic lightweight multifilament mesh was implanted onlay (placement of the mesh over the anterior abdominal wall into the subcutaneous tissue) to enforce the midline fascial closure. These three patients had a grade 2 hernia according the modified VHWG system; none of these three patients required ostomy closure. In these cases, the surgeon decided to enforce the midline because the incisional hernia was at least a second recurrence and the abdominal wall muscles were of poor quality. In 12 (20%) cases, a previously implanted and infected synthetic mesh was removed during the Permacol implant surgery. No patients required component separation. The mean operating time was 192  min (range 100–360  min). In all patients, two drains were inserted into the subcutaneous tissue and these were removed when the output was <50  mL/day.

### Surgical complications

SSO occurred in 21 (35%) patients (Table 2). These complications included: 14 (66.6%) patients with wound infections, five (23.8%) with seroma, one (4.7%) with skin necrosis and one (4.7%) with bulging. Most SSOs (57.1%) were grade 1–2 according to the Clavien-Dindo classification system. One patient with a wound infection died from pneumonia with respiratory failure. In one patient with minor wound dehiscence, the Permacol mesh was removed after transcutaneous migration of the implant. Here, the patient’s body did not incorporate the mesh, but rather created a thick fibrotic layer without a hernia defect; the mesh was rejected a little at time, until the surgeon decided to remove it. All wound infections were managed with advanced wound dressings and in four patients VAC (vacuum assisted closure) therapy was applied with good outcomes. VAC therapy was also used in patients with skin necrosis. Early post-operative complications occurred in 50% of patients with a BMI > 35, and 37.1% of patients with modified VHWG grade 3. The complication rate was significantly lower in patients classified as Chevrel W1–2 (7.6%) compared to those classified as Chevrel W3–4 (44.4%) *(p* = *0.011)* (Table 3).

**Table 2:** Post-operative complications.

	N (%)	Clavien-Dindo
Surgical Site Occurence	21 (35%)	
Wound infection	14 (66.6%)	1 Grade I
		5 Grade II
		7 Grade III
		1 Grade V
Seroma	5 (23.8%)	5 Grade I
Bulging	1 (5%)	1 Grade III
Skin necrosis	1 (5%)	1 Grade II

**Table 3:** Characteristics of patients with and without complications.

Characteristics	Patients with complications (n = 21)	Patients without complications (n = 39)
BMI > 35	8 (38%)	8 (20%)
Diabetes mellitus	6 (28.5%)	10 (25.6%)
Smoking status	7 (33.3%)	15 (38.5%)
COPD	6 (28.5%)	10 (25.6%)
Chevrel M1-M2	9 (42.9%)	14 (37.8%)°
Chevrel M3-M4	12 (57.1%)	23 (62.2%)°
Chevrel W1–2*	*1* (*4.8%)*	*13* (*34.2%)*^*§*^
Chevrel W3–4*	*20* (95.2%)*	*25* (*65.8%)*^*§*^
VHWG Grade 2	8 (38%)	13 (33.3%)
VHWG Grade 3	13 (61.9%)	22 (56.4%)
ASA II	9 (42.9%)	13 (33.3%)
ASA III-IV	12 (57.1%%)	26 (66.7%)
Bridge position	11 (52.4%)	17 (43.6%)
Underlay position	7 (33.3%)	16 (41%)
Sublay position	3 (14.3%)	6 (15.4%)
Fascial closure	10 (47.6%)	22 (56.4%)
Non-fascial closure	11 (52.4%)	17 (43.6%)

BMI: body mass index; COPD: Chronic Obstructive Pulmonary Disease; ASA: American Society of Anesthesiologists; VHWG: modified Ventral Hernia Working Group.

°Two missing data.

^§^One missing data.

*p = 0.011.

### Post-surgical follow-up

The mean hospital length-of-stay was 12.75 days (range 3–65 days); the mean hospital length-of-stay was significantly longer for patients with early complications (18 days vs. 10 days) (*p* < *0.05;* Mann-Whitney test). The 30-day mortality rate was 6.7% and was unrelated to the mesh implant. Cardiovascular complications were the cause of death in two patients, and pneumonia with respiratory failure was the cause of death in another two patients. The mean follow-up was 21.5 ± 17.11 months, but 10 patients (16.7%) were lost to follow-up. Excluding these 14 patients, the hernia recurrence rate in the remaining 46 patients was 32.6% (Table 4), with the majority (93.3%) recurrences occurring within 12 months. Recurrence was confirmed radiologically by CT scan. There were no statistically significant differences in the recurrence rate according to the surgical repair technique. Patients classified as Chevrel W3–4 were more likely to develop a recurrent hernia than patients classified as Chevrel W1–2 (40.0% versus 9.1%); however, this difference between the two groups was not statistically significant (*p* *= 0.07*). Finally, 53.3% of patients who developed complications presented with a recurrent hernia within 24 months of surgery.

**Table 4:** Recurrence rate and characteristics of 46 patients with long-term follow-up.

Recurrences at 24 months	15 (32.6%)	
Recurrences at 12 months	13 (28.2%)	
**Characteristics**	**Patients with recurrences (n = 15)**	**Patients without recurrences (n = 31)**
BMI > 35	6 (40%)	8 (25.8%)
Diabetes mellitus	3 (20%)	8 (25.8%)
Smoking status	9 (60%)	9 (29%)
COPD	4 (26.7%)	8 (25.8%)
Chevrel M1-M2	7 (46.7%)	9 (30%)°
Chevrel M3-M4	8 (53.3%)	21 (70%)°
Chevrel W1–2	*1* (*6.7%)*	*10* (*32.3%)*
Chevrel W3–4	*14* (*93.3%)*	*21* (*67.7%)*
VHWG Grade 1	1 (6.7%)	3 (9.7%)
VHWG Grade 2	6 (40%)	10 (32.3%)
VHWG Grade 3	8 (53.3%)	18 (58%)
ASA II	6 (40%)	8 (25.8%)
ASA III-IV	10 (66.7%)	23 (74.2%)
Bridge position	9 (60%)	12 (38.7%)
Underlay position	4 (26.7%)	13 (41.9%)
Sublay position	2 (13.3%)	6 (19.4%)
Fascial closure	6 (40%)	19 (61.3%)
Non-fascial closure	9 (60%)	12 (38.7%)
**Early post-operative complications**	8 (53.3%)	10 (32.3%)
Wound infection	7 (46.7%)	7 (22.6%)
Seroma	1 (6.7%)	1 (3.2%)
Skin necrosis	0 (0.0%)	1 (3.2%)
Bulging	0 (0.0%)	1 (3.2%)
Rejection	0 (0.0%)	1 (3.2%)

BMI: body mass index; COPD: Chronic Obstructive Pulmonary Disease; ASA: American Society of Anesthesiologists; VHWG: modified Ventral Hernia Working Group.

°Two missing data.

## Discussion

Biological meshes have been used for abdominal surgery for ~20 years, and their use is mainly dictated by the type of surgical wound. Some studies have suggested that a biological mesh can better resist infections than a synthetic mesh, and are thus recommended for use in contaminated fields or in patients with VHWG grade 3^23^. Data from a recent meta-analysis that compared the outcomes from biological versus synthetic meshes in contaminated wounds, however, showed that a biological mesh is not superior to a synthetic mesh in terms of SSO or recurrences^27^. In our opinion, the evidence from this meta-analysis is weak due to the poor quality of the studies included. Patients with CAWD typically present with associated problems due to comorbidities, contaminated wounds, fistulas or synthetic mesh infection. In these cases, the surgical repair procedure is extremely difficult and complex. For this reason, we consider that using a biological mesh for CAWD repair at least avoids the problems and risks directly incurred by a synthetic mesh.

A literature review on biological meshes found that Permacol is one of the most studied mesh types in the published literature^28^, especially in the context of CAWD repair. These studies, however, are mainly retrospective, single institution case series^5,9,12–14,28–34^ with only three multicenter retrospective studies^3,15,22^ and one cross-sectional study^21^ published to date. Our series of 60 patients represents the first large, multi-centre Italian study on Permacol mesh implants in patients with CAWD. We found that most patients had more than one comorbidity that was not well compensated, which explains why 63.3% of our patients had a high ASA score. Consistent with a previous study^3^, the ASA score did not significantly impact on the incidence of early complications or hernia recurrence.

In 2010, the VHWG proposed a grading system for SSO risk and concluded that a biological implant should be considered in patients with a potential risk of infection (grade 2–3), and preferred in high risk patients (grade 4)^6^. Many analyses of patients with CAWD have found that most patients are at high SSO risk, with a VHWG grade >2. For example, Chand *et al*. performed a retrospective analysis of 343 patients with a Permacol surgical implant, including 213 incisional and 130 ventral hernias. Most of these patients (82.9%) were categorized as VHWG grade 2 or 3^3^. A Dutch cohort study included 77 patients with CAWD who were all classified as VHWG grade 3–4^21^. Finally, a multi-centre French audit study recruited 250 patients with VHWG grade 3–4^22^. In our study, 93.3% of the patients were modified VHWG grade 2–3, reflecting the high rate of comorbidities and CAWDs with a contaminated surgical field. In patients with VHWG grade 1 (6.7%), a biological mesh was indicated due to the presence of a defect >10  cm, multiple previous repair attempts or multiple defects, as indicated by CAWD characteristics^5^.

The surgical technique can have an impact on clinical outcomes. Giordano *et al*. found that independent of the mesh position, fascial closure is the only significant factor that affects hernia recurrence, with a higher recurrence rate when closure is not achieved (18.2% versus 5.4%)^5,15^. In our cohort, we achieved fascial closure in 53.3% patients, but this did not significantly impact the complication or recurrence rate (despite the fascia not being closed in 52.4% patients with complications and 60% of patients with recurrences). In our case series, no patients required component separation. This finding might be because the technique has only recently been implemented in our practice for CAWD repair, and the technique extends the complexity of an already challenging surgical procedure. Over recent years, a biosynthetic mesh to repair a CAWD is often considered in alternative to biological mesh, when a complete fascial closure is achieved with anterior or posterior component separation with or without transversus abdominis release (TAR) technique.

During our patient enrolment period (2010–2016) most of the participating surgeons widely used the bridging technique and the component separation technique was rarely applied in combination with a biological mesh. Our practice has changed over recent years, with a preference for underlay and sublay mesh placement even in combination with the component separation technique. The bridge technique is associated with an increased recurrence rate. Bridging defects with a biological mesh leads to stretching and laxity and can, therefore, result in failure of the repair, or at least in a bulging^5,29^. A previous study found that when Permacol was used to bridge a fascial defect, the hernia recurrence rates were >80%^15^. Here, we found that only 32.1% patients encountered a recurrence 24 months after bridging repair with Permacol. Only one patient developed a bulging. We did not consider a bulge as a recurrence because, even if the midline is not closed over the biological mesh, there is no interruption between the mesh and the abdominal wall layer^21,25^. Rather, we consider that for patients requiring CAWD repair, the onset of a postoperative bulging or abdominal wall weakness is an acceptable result and does not represent complete failure of the procedure.

The Permacol collagen matrix promotes new collagen deposition and thus confers biocompatibility and immunogenic ability that promotes infection resistance^6,7,10^. With regards to the risk of infection and the complexity of the cases in our series, we found that 35% of patients developed a complication, mainly either a wound infection (23.3%) or seroma (8.3%). The onset of complications significantly prolonged the hospital length-of-stay. A previous large series reported a high complication rate of 40.5%, with 19% seromas, 15.2% wound infections and 3.2% hematomas occurring after Permacol implantation^3^. These rates have been confirmed by a recent multicenter audit that reported an SSO rate of 48.8% consisting mainly of abscesses (66.4%)^22^. Only 29.6% cases in this series required a repeat intervention; these cases were mostly due to intraperitoneal complications (31.1%), wound abscesses (36.5%), seroma (10.8%), hematoma (12.2%) or wound dehiscence (9.5%)^22^. Such complications, however, can be readily managed by bedside incision and drainage and oral antibiotics^20,21^. Similar to previously published studies^22^, we found that the defect size, expressed by the Chevrel classification in width (W), influenced the rate of post-operative complications. Defects >10  cm typically require greater mobilization of the muscolofascial units; here, tissue microvascularization must be sacrificed and a third space must be created. This process tends to be associated with a higher rate of post-operative infections. Finally, biological mesh removal has been reported in case studies where an infection with mesh disruption has occurred^21,22^; only rarely has a biological mesh been removed because of rejection^29,35,36^. In our series, the mesh was removed in only one case.

Using Permacol as a crosslinker increases graft stability and durability, even in contaminated fields. Here, the hernia recurrence rate at 12 months is lower (6.6%) than the recurrence rate when using a non-cross-linked porcine mesh (21.2%)^28^. In our study, the overall hernia recurrence rate was 25% at 24 months follow-up. The majority of these affected patients (93.3%) had already presented with a hernia recurrence at 12 months. Although the wound class has been suggested to increase the risk of hernia recurrence following CAWD repair^5^, we found no such effect on the recurrence rate in our series. Our data are consistent with the published literature^5,13–15,33^, especially when comparing our findings to studies where a long-term follow-up of at least 24 months was achieved. Further studies are now needed to identify the risk factors for hernia recurrence and complications, using multivariate analyses.

## Conclusions

The high cost of Permacol means that it cannot be recommend for routine abdominal wall reconstruction^37^. As such, some clinicians only support this type of mesh for use in CAWD repair^5,7,15^. Unfortunately, the data regarding the superiority of using a biological mesh versus a synthetic mesh for CAWD repair are poor. Randomized studies in the context of CAWDs are difficult to perform due to a lack of uniformity in CAWD classification, the various possible surgical techniques that can be performed and high patient heterogeneity. For this reason, some clinicians do not support the use of a biological mesh for CAWD repair until more conclusive data are available^27,38,39^.

Our study represents the first large multi-centre Italian study on Permacol implantation in patients with CAWD. The use of biological meshes has increased rapidly over the past 10 years, but high-level evidence is lacking to demonstrate superiority. Our study, although retrospective, supports the use of Permacol in CAWD repair. Consistent with previous reports^5,15,16,21,22^, we propose that Permacol is safe and feasible, and show that it elicits an acceptable early SSO and long-term recurrence rate. This multicenter experience using Permacol should encourage surgeons who encounter challenging surgical situations like CAWDs to use a biological implant such as Permacol; continued use of Permacol will help grow the evidence base. Future multicenter studies that compare the different types of biological and biosynthetic mesh are now required. In addition, a long-term follow-up is now warranted to better evaluate the utility of biological mesh implants for abdominal wall repair in high-risk patients.

## References

[1] Patel NG, Ratanshi I, Buchel EW (2018). The Best of Abdominal Wall Reconstruction. Plast. Reconstr. Surg..

[2] Burger JWA (2004). J Long-term follow-up of a randomized controlled trial of suture versus mesh repair of incisional hernia. Ann. Surg..

[3] Chand B (2014). A retrospective study evaluating the use of Permacol surgical implant in incisional and ventral hernia repair. Int. J. Surg..

[4] Slater NJ (2014). Criteria for definition of a complex abdominal wall hernia. Hernia.

[5] Limura E, Giordano P (2017). Biological implant for complex abdominal wall reconstruction: a single institution experience and review of literature. World J. Surg..

[6] Breuing K (2010). Incisional ventral hernias: review of the literature and recommendations regarding the grading and technique of repair. Surgery.

[7] Darehzereshki A (2014). Biologic versus nonbiologic mesh in ventral hernia repair: a systematic review and meta-analysis. World J. Surg..

[8] Harper Catherine (2001). Permacol™: clinical experience with a new biomaterial. Hospital Medicine.

[9] Hsu PW (2009). Evaluation of porcine dermal collagen (Permacol) used in abdominal wall reconstruction. J. Plast. Reconstr. Aesthet. Surg..

[10] Rosen MJ (2010). Biologic mesh for abdominal wall reconstruction: a critical appraisal. Am. Surg..

[11] Latifi R (2017). Risk-adjusted adverse outcomes in complex abdominal wall hernia repair with biologic mesh: A case series of 140 patients. Int. J. Surg..

[12] Cheng AW, Abbas MA, Tejirian T (2013). Outcome of abdominal wall hernia repair with Permacol biologic mesh. Am. Surg..

[13] Abdelfatah MM, Rostambeigi N, Podgaetz E, Sarr MG (2015). Long-term outcomes (5-year follow-up) with porcine acellular dermal matrix (Permacol) in incisional hernias at risk for infection. Hernia.

[14] Iacco A, Adeyemo A, Riggs T, Janczyk R (2014). Single institutional experience using biological mesh for abdominal wall reconstruction. Am. J. Surg..

[15] Giordano P., Pullan R. D., Ystgaard B., Gossetti F., Bradburn M., McKinley A. J., Smart N. J., Daniels I. R. (2015). The use of an acellular porcine dermal collagen implant in the repair of complex abdominal wall defects: a European multicentre retrospective study. Techniques in Coloproctology.

[16] Atema JJ (2017). Major complex abdominal wall repair in contaminated fields with use of a non cross-linked biologic mesh: a dual institutional experience. World J. Surg..

[17] Harth KC (2013). Biologic mesh use practice patterns in abdominal wall reconstruction: a lack of consensus among surgeons. Hernia.

[18] Harris HW (2013). Biologic mesh for ventral hernia repair: a cautionary tale. Ann. Surg..

[19] Petter-Puchner AH, Dietz UA (2013). Biological implants in abdominal wall repair. Br. J. Surg..

[20] Majumder A (2016). Comparative analysis of biologic versus synthetic mesh outcomes in contaminated hernia repairs. Surgery.

[21] Kaufmann R (2019). Repair of complex abdominal wall hernias with a cross-linked porcine acellular matrix: cross-sectional results of a Dutch cohort study. Int. J. Surg..

[22] Doussot A (2019). Indications and outcomes of a cross-linked porcine dermal collagen mesh (Permacol) for complex abdominal wall reconstruction: a multicentre audit. World J. Surg..

[23] Berger RL (2013). Development and validation of a risk-stratification score for surgical site occurrence and surgical site infection after open hernia repair. J. Am. Coll. Surg..

[24] Korenkov M (2001). Classification and surgical treatment of incisional hernia. Results of an experts’ meeting. Langenbecks Arch. Surg..

[25] Garcia-Urena MA (2019). Abdominal wall reconstruction utilizing the combination of absorbable and permanent mesh in a retromuscolar position: a multicentre prospective study. World J. Surg..

[26] Clavien PA (2009). The Clavien-Dindo classification of surgical complications: five years experience. Ann. Surg..

[27] Atema JJ, de Vries FE, Boermeester MA (2016). Systematic review and meta-analysis of the repair of potentially contaminated and contaminated abdominal wall defects. Am. J. Surg..

[28] Trippoli S (2018). Biological meshes for abdominal hernia: Lack of evidence-based recommendations for clinical use. Int. J. Surg..

[29] Giordano S, Garvey PB, Baumann DP, Liu J, Butler CE (2017). Primary fascial closure with biologic mesh reinforcement results in lesser complication and recurrence rates than bridged biologic mesh repair for abdominal wall reconstruction: A propensity score analysis. Surgery.

[30] Sarmah BD, Holl-Allen RTJ (1984). Porcine dermal collagen repair of incisional herniae. Br. J. Surg..

[31] Parker DM, Armstrong PJ, Frizzi JD, North JH (2006). Porcine dermal collagen (Permacol) for abdominal wall reconstruction. Curr. Surg..

[32] Catena F (2007). Use of porcine dermal collagen graft (Permacol) for hernia repair in contaminated fields. Hernia.

[33] Shaikh FM, Giri SK, Durrani S, Waldron D, Grace PA (2007). Experience with porcine acellular dermal collagen implant in one-stage tension-free reconstruction of acute and chronic abdominal wall defects. World J. Surg..

[34] Connolly PT (2008). Outcome of reconstructive surgery for intestinal fistula in the open abdomen. Ann. Surg..

[35] Loganathan A, Ainslie WG, Wedgwood KR (2010). Initial evaluation of Permacol bioprosthesis for the repair of complex incisional and parastomal hernias. Surgeon.

[36] Wotton FT, Akoh JA (2009). Rejection of Permacol mesh used in abdominal wall repair: A case report. World J. Gastroenterol..

[37] Bellows CF, Smith A, Malsbury J, Helton WS (2013). Repair of incisional hernias with biological prosthesis: a systematic review of current evidence. Am. J. Surg..

[38] Köckerling F (2018). What is the evidence for the use of biologic or biosynthetic meshes in abdominal wall reconstruction?. Hernia.

[39] Tampaki EC, Tampakis A, Kontzoglou K, Kouraklis G (2017). Commentary: Evidence for replacement of an infected synthetic by a biological mesh in abdominal wall hernia repair. Front. Surg..

